# Propranolol Restricts the Mobility of Single EGF-Receptors on the Cell Surface before Their Internalization

**DOI:** 10.1371/journal.pone.0083086

**Published:** 2013-12-09

**Authors:** Carolina Otero, Max Linke, Paula Sanchez, Alfonso González, Iwan A. T. Schaap

**Affiliations:** 1 Center for Integrative Medicine and Innovative Science (CIMIS), Universidad Andres Bello, Santiago, Chile; 2 Centro para el Desarrollo de la Nanociencia y Nanotecnologia, Santiago, Chile; 3 III. Physikalisches Institut, Faculty of Physics, Georg-August Universität, Göttingen, Germany; 4 Departamento de Inmunología Clínica y Reumatología, Facultad de Medicina and Centro de Envejecimiento y Regeneración, Departamento de Biología Celular y Molecular, Facultad de Ciencias Biológicas, Pontificia Universidad Católica de Chile, Santiago, Chile; 5 Center for Nanoscale Microscopy and Molecular Physiology of the Brain (CNMPB), Göttingen, Germany; Thomas Jefferson University, United States of America

## Abstract

The epidermal growth factor receptor is involved in morphogenesis, proliferation and cell migration. Its up-regulation during tumorigenesis makes this receptor an interesting therapeutic target. In the absence of the ligand, the inhibition of phosphatidic acid phosphohydrolase activity by propranolol treatment leads to internalization of empty/inactive receptors. The molecular events involved in this endocytosis remain unknown. Here, we quantified the effects of propranolol on the mobility of single quantum-dot labelled receptors before the actual internalization took place. The single receptors showed a clear stop-and-go motion; their diffusive tracks were continuously interrupted by sub-second stalling events, presumably caused by transient clustering. In the presence of propranolol we found that: i) the diffusion rate reduced by 22 %, which indicates an increase in drag of the receptor. Atomic force microscopy measurements did not show an increase of the effective membrane tension, such that clustering of the receptor remains the likely mechanism for its reduced mobility. ii) The receptor got frequently stalled for longer periods of multiple seconds, which may signal the first step of the internalization process.

## Introduction

The epidermal growth factor receptor (EGFR) is one of the best-characterized receptor (protein) tyrosine kinases in endocytic trafficking. This transmembrane receptor has a key role in organ morphogenesis, maintenance and repair. EGFR is also involved in various cancers by contributing to malignancy due to over-expression or oncogenic mutations [1]. Thus EGFR is a target for therapeutic intervention in different cancer treatments [2]. Endocytic trafficking and signalling of EGFR, as well as of other signalling receptors, are tightly intertwined [3,4] and cancerous cells take advantage of such a functional link to enhance the oncogenic influence of EGFR [5]. This motivates research to increase our understanding of these processes at the molecular level. 

For a long time the spatiotemporal dynamics of EGFR at the cell surface, before its internalization have been attracting great interest [6] but still remain incompletely understood. A single EGFR on the plasma membrane binds EGF to form a pre-signalling state after which a second EGFR binds forming a homo-dimer with the first one. Although experimental evidence for EGFRs in solution is consistent with a ligand-induced allosteric dimerization model [6], there is increasing evidence that cell surface EGFR activation depends on conformational changes within preformed dimers, interactions between receptor dimers, heterodimerization, cross-talk with other receptor types, and ligand-independent lateral propagation of the activation processes, [7,8]. Single molecule studies have also identified a signal amplification mechanism involving the dynamic clustering of the EGFR [9]. The dimer becomes then auto-phosphorylated and later internalized. This starts the signalling cascade [8,10].

Since EGFR has a key role in cancer, it is important to understand how drugs affect the availability and functionality of EGFR in the cell membrane. In this study we focus on propranolol, an inhibitor of phosphatidic acid (PA) phosphohydrolase activity, which leads to increased PA levels in the cell [11-13]. Although propranolol does not interact with EGFR directly, it leads to an accumulation of PA accompanied by a stimulation of type 4 phosphodiesterases (PDE4) and a significant decrease in both cAMP levels and protein kinase-A (PKA) activity. This provokes a reversible redistribution of empty EGFR from the cell surface to recycling endosomes through PA-mediated signalling toward the PDE4/cAMP/PKA pathway, thus reducing the receptor accessibility for external stimuli [14,15]. Both clathrin-dependent and clathrin-independent pathways seem to be involved in this PA-mediated pathway [16]. The molecular mechanisms that underlie such receptor internalization under propranolol treatment remain unknown.

In order to obtain more insight in the events that precede the internalization process we investigated if propranolol affects the mobility of the receptors before they get internalized. The two extreme behaviours that are expected are the unhindered diffusion of the receptor along the cell membrane, and the receptor getting stuck at a fixed location. In addition, any intermediate behaviour, showing a combination of both, can occur when the receptor is hindered in its motion. Either by binding partners or aggregates in the lipid bilayer [17], or by interaction with protein networks such as the f-actin or clathrin [18,19].

To be able to follow the sequence of events of individual EGFR we tracked the motion of single fluorescently labelled receptors while they moved along the cell surface. As label we used quantum dots (QD) that were coupled to the EGFR antibodies. QDs are nanometre sized semiconductors that, similar to conventional fluorophores, can emit photons upon being exited with light. In contrast to conventional fluorophores, QDs have a higher photo-stability, which makes them ideal probes for long-term single particle tracking and time-lapse-microscopy [20]. Quantum dots do however occasionally blink [21] which can complicate single particle tracking in a densely labelled environment. To avoid this, we labelled only a subset of EGFR.

To obtain a fluorescent signal only from the cell periphery we used total internal reflection fluorescence (TIRF) microscopy. In this technique the excitation light does not enter into the sample but is reflected off the coverslip. At the coverslip-buffer interface an evanescent wave will penetrate into the sample for about 200 nm [22]. Thus only fluorophores that are close to the coverslip are excited and background fluorescence coming from within the cell is largely avoided.

We observed that the mobility of EGFR is dominated by rapid alternations between diffusive and non-diffusive, which originates from a stop-and-go motion of the receptor. In the presence of propranolol we did observe a significant reduction in the average diffusion constant, but no increase in the stop-and-go ratio. We performed atomic force microscopy (AFM) measurements to exclude the possibility that the reduced receptor mobility was caused by an increase of the rigidity of the membrane or its interaction with the cell cortex [23]. Also we found that propranolol eventually induced stalling of EGFR for multiple seconds, which we speculate to be the preparing step for the respective drug induced endocytosis. 

## Results

**Figure 1:**
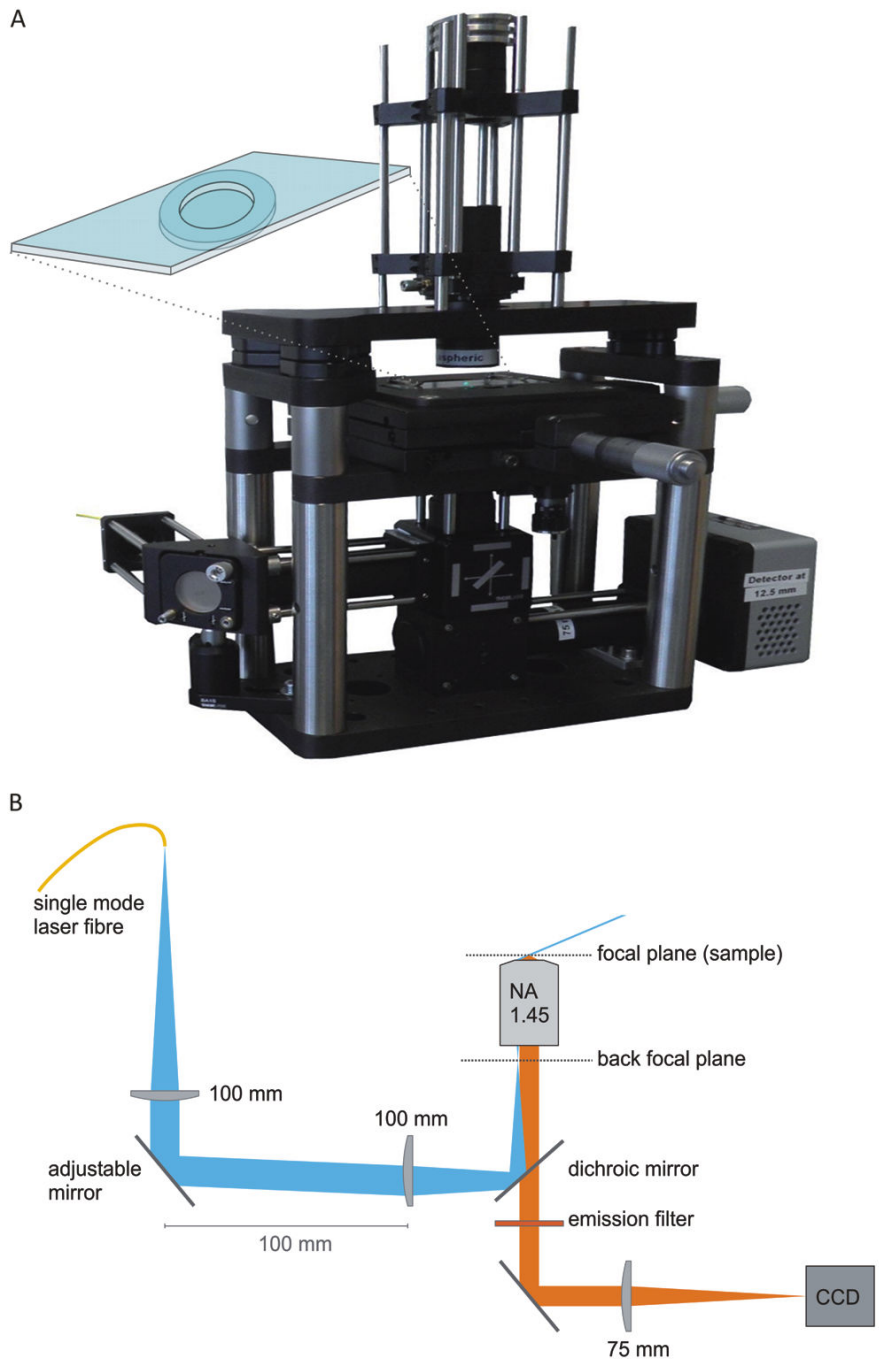
Minimal design inverted TIRF microscope. a) A photograph of the instrument, the view on the objective is hidden by the x-y sample stage. The inset cartoon shows the open-top sample chamber. b) A cross-sectional drawing of the essential components of the instrument. The TIRF objective is mounted on a flexure z-stage to adjust the focus. The image is directed via a mirror and tube-lens onto an EM-CCD camera. The excitation laser light comes from a single-mode optical fibre and is coupled into the optical path via a dichroic mirror. The TIRF angle is adjusted by adjusting the angle of the second mirror that is placed in a plane that is conjugate to the back focal plane of the objective. The sample is mounted on a mechanical xy stage and illuminated from top via a small illumination tower that is set-up according to the Köhler illumination scheme.

To be able to perform single molecule tracking experiments we constructed an inverted microscope around a commercial TIRF microscope objective. Due to its minimal and rigid design (Figure 1) this instrument offers a low focus drift which is required for observation times of minutes at diffraction limited performance. An additional benefit of this simple design is that the building costs are mainly determined by the TIRF objective and EM-CCD camera. The total cost of approximately 30.000 euro shows that single molecule tracking experiments are also within reach for laboratories with a limited budget. To be able to administer drugs during imaging we used an open-top sample chamber. The coverslip containing the cells was glued under a microscope slide that had a 1 cm diameter opening cut out (see methods and inset Figure 1A).

**Figure 2:**
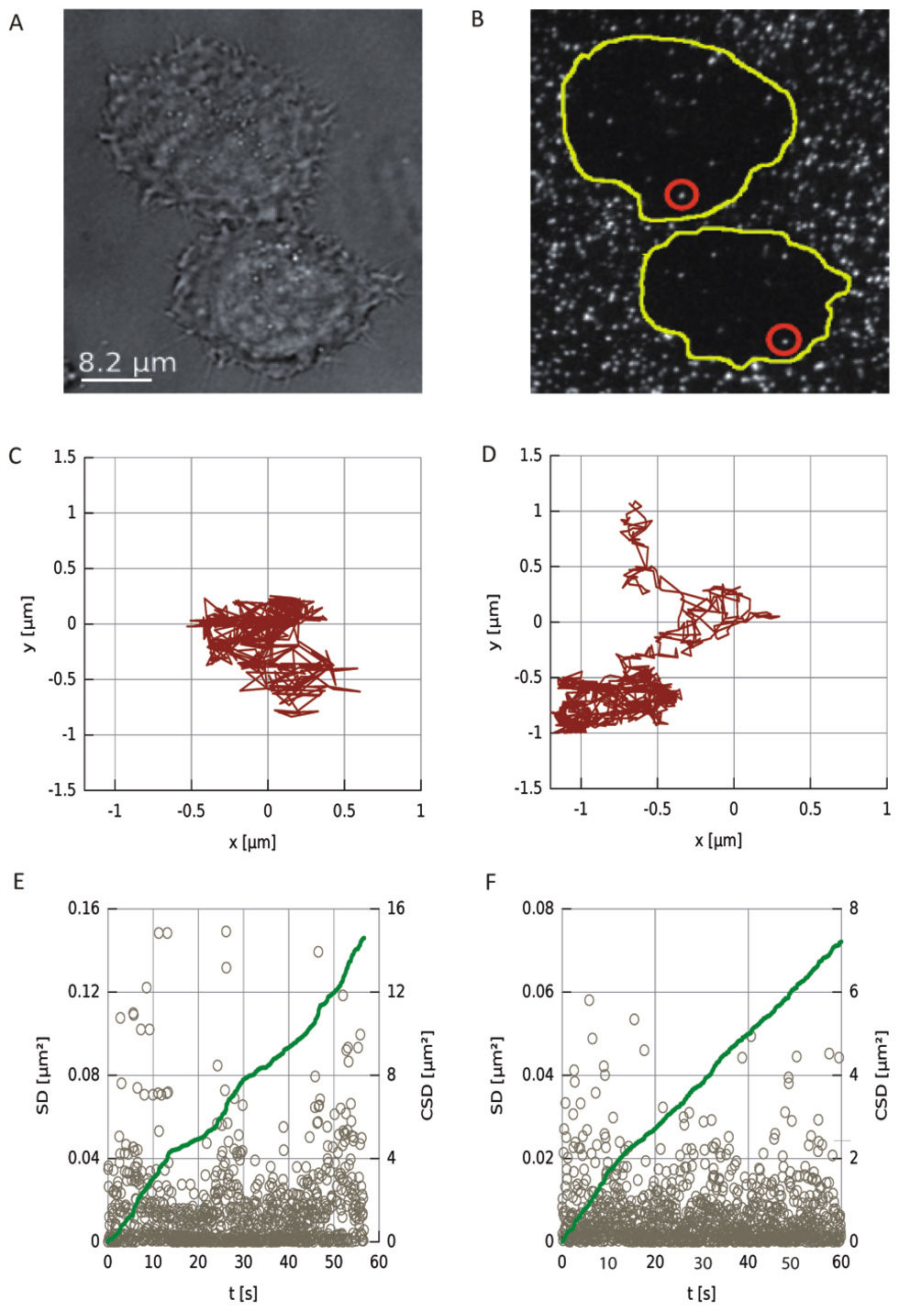
Tracking single EGFR. a) The bright-field and b) fluorescence image of two cells. On the cells typically multiple mobile QDs were visible. The coverslip is covered with immobilized QDs. c, d) Two typical trajectories of single EGFR recorded over one minute. e, f) The corresponding SD/CSD-plots. The SD steps between successive frames are shown in grey and the cumulative plot in green. In some curves apparent changes in slope could be observed (c), but the majority of CSD plots looked like (d) without obvious variations in slope.

To follow the response of single EGFR upon the addition of propranolol we used a single molecule tracking approach. A small fraction of individual EGFR molecules was tagged with a conjugate of one QD linked to one anti-EGFR antibody Fab fragment. In [24] it was shown that this QD conjugate had no effect on the EGF induced phosphorylation of the receptor thus suggesting an unperturbed receptor function. Because of the use of single Fab fragments (instead of whole antibodies) the QD conjugate is not expected to induce dimerization of EGFR, which makes this an ideal method to study the dimerization of monomeric receptors [24,25]. Single labelled EGFRs could be imaged by our TIRF microscope at high signal to noise ratio (Figure 2a and b). The motion of the QDs was quantified using a tracking algorithm based on the centre of intensity algorithm [26] (see methods).

We have monitored the squared displacements (SD) between successive frames and summed these to obtain the cumulative square distance (CSD) as function of the time. Basically the total length of the sampled path of the QD is thus quantified. The advantage of this approach, as compared to the mean square distance (MSD, which shows the squared distance from the starting point), is that the CSD is not affected by boundary conditions that could limit the area available for diffusion. As result the CSD gives a good estimate for the average diffusion coefficient and largely ignores effects of spatial constraints of the diffusion area. Figure 2 (c and d) shows typical paths of single receptor diffusion obtained via this tracking routine. Figure 2 e and f show the individual (squared) displacements between frames (grey circles). The respective CSD curves are represented in green in Figure 2 (e and f). 

**Figure 3:**
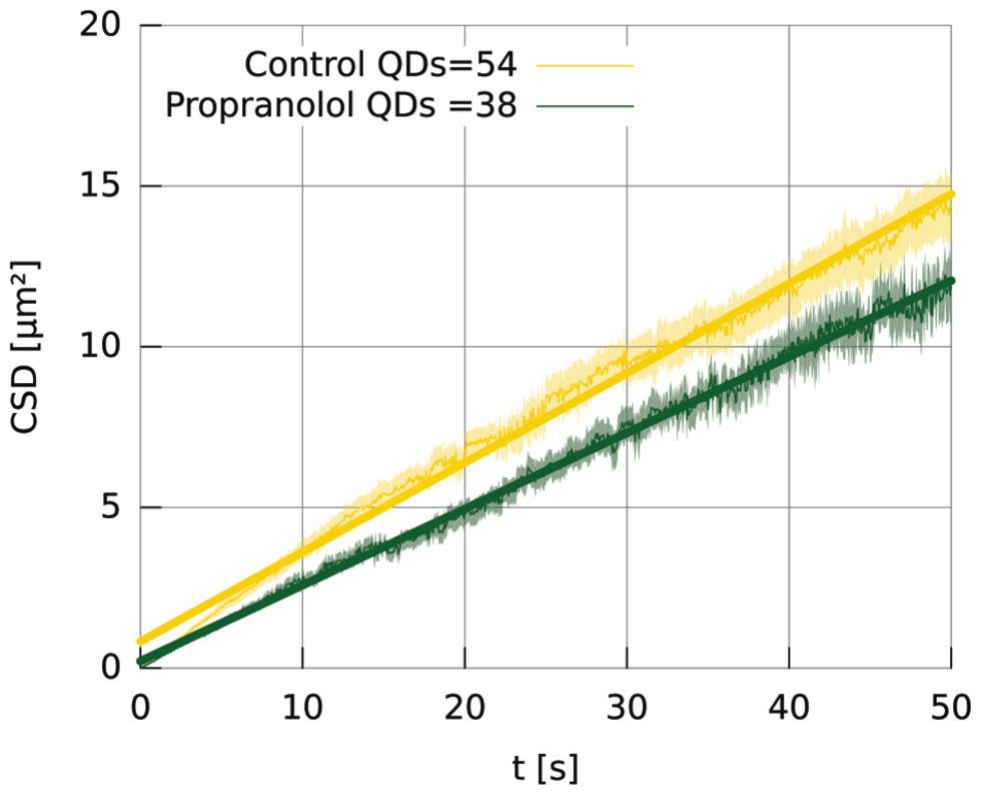
Average CSD plots with and without propranolol. A total of 92 individual EGFR tracks of at least 1 minute were used to calculate the average CSD curves. The average diffusion coefficient was obtained by a linear fit to the curves and give for Dcontrol= 0.077 ± 0.002 µm^2^/s and Dpropranolol= 0.060 ± 0.002 µm^2^/s (mean ± s.e.m.). To obtain the s.e.m., the CSD of each individual track was fitted. The s.e.m. was calculated from these individual fits.

To test if propranolol had an effect on the average diffusion constant, HeLa cells were incubated with or without propranolol, and the motion of single EGFR was quantified as described above. Figure 3 shows the averaged CSD plots for a total of 92 particles that were measured in 11 different experiments. We found a 22 % reduction of the diffusion coefficient when propranolol was added: Dcontrol= 0.077 µm^2^/s and Dpropranolol= 0.060 µm^2^/s. 

**Figure 4:**
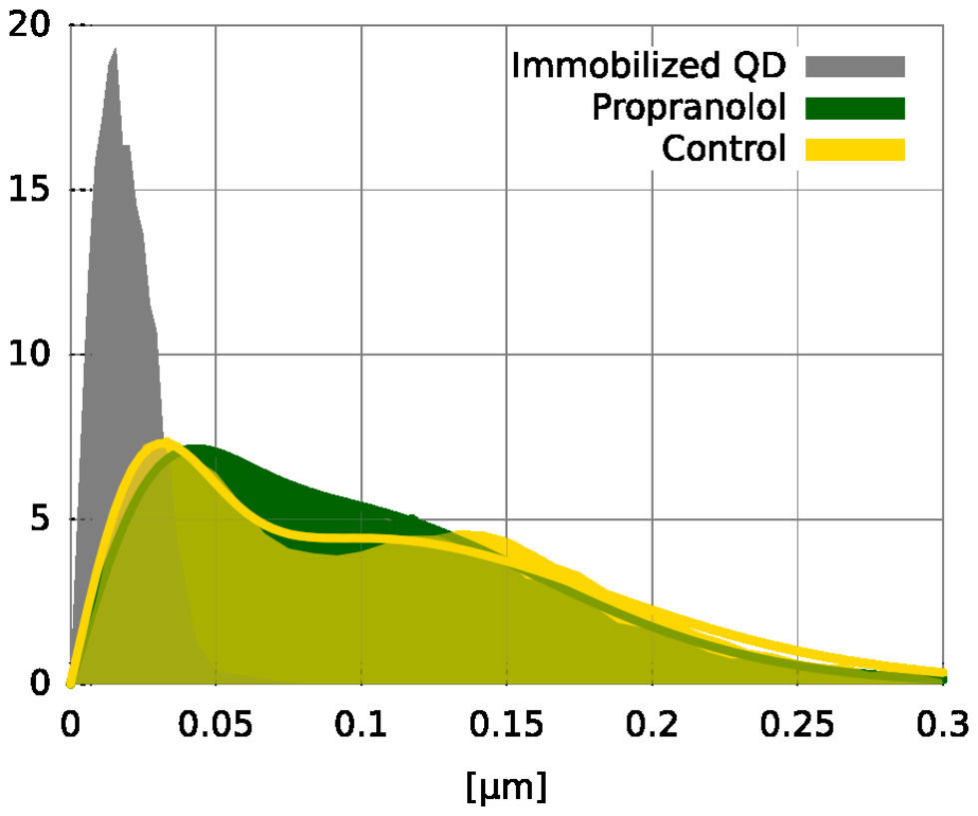
Step-size distribution with and without propranolol. The histograms include all individual displacements (steps) between the successive frames of all tracked EGFR without (yellow) and with propranolol (green). The coloured histograms each show two peaks, representing two populations of step-sizes. Control experiments performed with immobilized QDs (grey) shows that the first peak represents particles that are almost stationary, the second peak shows the actual diffusive motion. The ratio between the areas of both peaks remains almost identical with and without propranolol. In presence of propranolol the diffusive peak has clearly shifted to lower step-sizes.

When looking at the individual SD-steps such as shown in Figure 2 (e and f, grey circles), it appears that many of these SD-steps were nearly zero whereas others showed relative large steps. In presence of propranolol the same phenomenon is visible, which leads to the question if EGFR exists in two states, a slow one and a fast one. When two distinct diffusion constants are present then one would also expect to see two populations when all step-sizes between successive frames of all curves are plotted in a histogram. Figure 4 shows, indeed, clearly 2 peaks in the histograms. From the comparison with the step-size histogram from immobilized quantum dots, it becomes obvious that the first peak corresponds to particles that are almost stationary. Only the second peak shows the actual diffusive behaviour. For the experiments with propranolol this second peak is shifted towards a lower step-size as compared to the control experiments, consistent with the reduced average diffusion constant as shown in Figure 3. 

The presence of two clearly distinguished peaks in the EGFR step-size histograms indicates that the receptors can exist in both a mobile and a semi-stationary state. These states exist both with and without propranolol. An investigation of all individual CSD tracks, such as shown in Figure 2 (E and F, green curve) did not reveal an obvious separation of stationary and mobile phases; the stationary phases last typically only between 1 and 5 frames. From this we conclude that the stationary states have only a very short lifetime, on the order of 100 ms. Physically, this can be understood by EGFR getting stuck continuously, but only for short amounts of time. This possibility is included in the cell membrane ’picket’-model [7,27]. 

Since we obtained the average diffusion constant by a fit to the CSD plots (Figure 3) this includes also the short lived sticking events which leads to an underestimation of the unrestricted diffusion speed. To obtain an estimate of the diffusion speed when the receptors are not hindered in their motion, we fitted a probability density function to the histograms of Figure 4 (see methods). From the 'fast' step-size peaks this gives a diffusion coefficient of: Dcontrol = 0.11 µm^2^/s and Dpropanolol = 0.084 µm^2^/s (table 1). The 24 % reduction of the diffusion coefficient in the presence of propranolol is identical with the observed reduction of the average diffusion coefficient measured with the CSD. Because the ratio between both peaks remains almost similar, propranolol mainly reduces the actual unrestricted diffusion speed but it does not increase the time the receptor spends in the semi-stationary phase.

**Table 1:** The fitted parameters (± s.d. of the fit) for equation 1 for the different step-size histograms.

	*C*	*D, slow*	*D, fast*
control	0.240 ± 0.007	0.0073 ± 0.0003	0.110 ± 0.002
propranolol	0.204 ± 0.004	0.0102 ± 0.0003	0.0835 ± 0.0009

**Figure 5:**
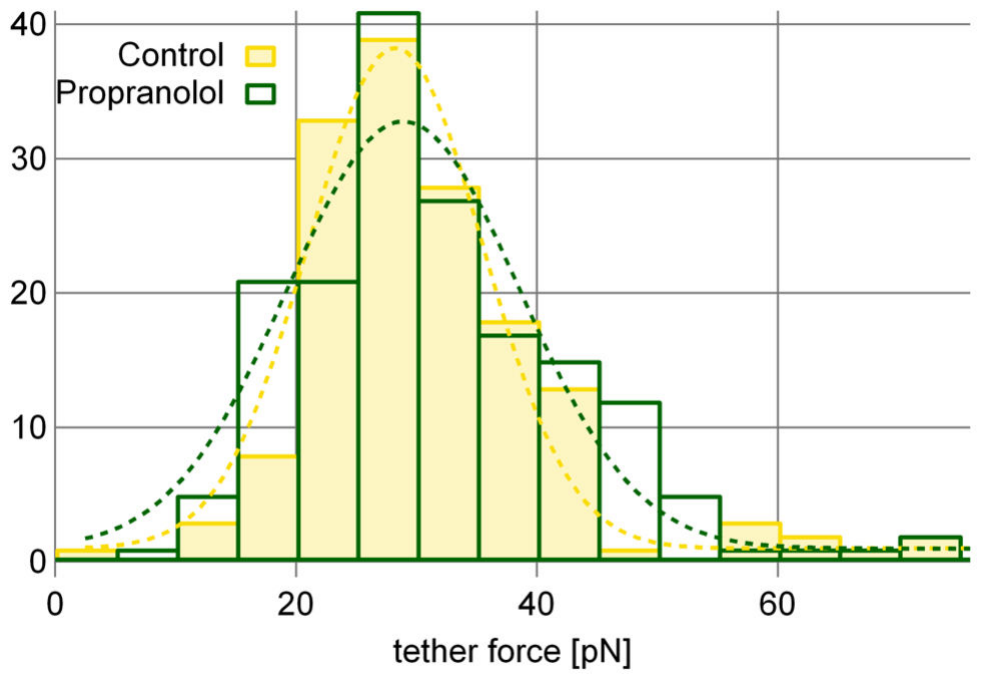
Propranolol does not affect the effective membrane tension. A total of 333 membrane tethers were pulled off 23 different cells, on 5 different cover slips using 4 different AFM cantilevers. The tether force is around 28 pN without a statistical relevant difference with and without propranolol. Inset) To measure the effective membrane tension an AFM tip was brought in contact with the cell and then pulled away. The rupture of the extracted membrane from the tip is visible as a sudden jump to zero force.

The reduction of the unrestricted diffusion coefficient in presence of propranolol can be caused by dimerization or clustering of EGFR but also by an increased rigidity of the plasma membrane. To test for changes in the composition of the plasma membrane or the underlying cell cortex we measured the effective membrane tension in absence and presence of propranolol. This was performed by extracting membrane tethers from the cell by AFM pulling experiments (inset Figure 5). The force that is required to extract a membrane tether depends on the membrane bending rigidity and on the interaction of the membrane with the cell cortex [28]. Such tether extraction measurements have been successfully applied to measure the effects of compounds like cholesterol and Latrunculin-A on the effective membrane tension [29,30]. Figure 5 shows that the tether force without propranolol (28.26 ± 0.83 pN) remains unaffected after the addition of propranolol (28.92 ± 1.02 pN). Propranolol does not induce measurable changes in the mechanical organization of the cell cortex and plasma membrane which could hinder the mobility of EGFR. 

**Figure 6:**
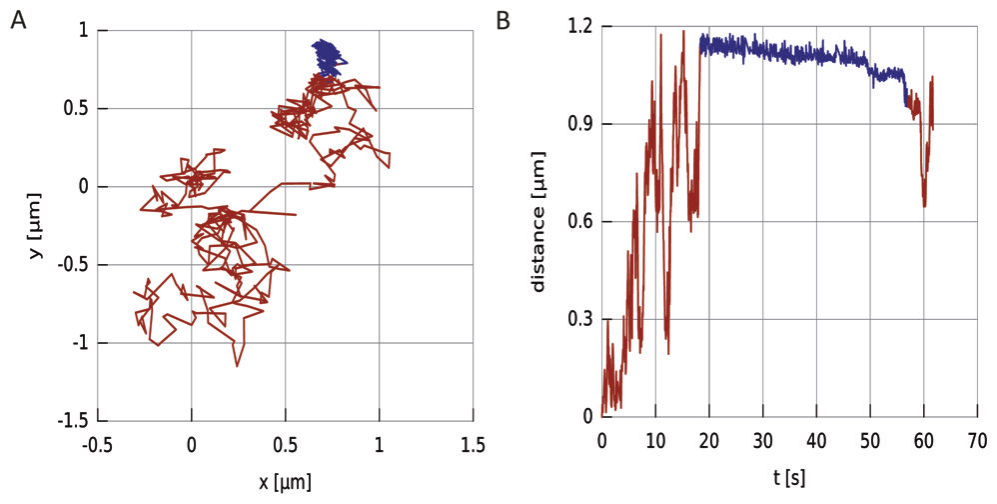
Propranol induces stalling of EGFR. a) Trajectory of an EGFR after propranolol treatment. After some time the receptor got stuck and remained at one location (indicated in blue). b) The same trajectory plotted against time. The stationary part (blue) is clearly distinguishable. After being stationary for almost 40 s the receptor continued its travel again.

Before, we showed that diffusive tracks of EGFR are a continuous alternation of stop-and-go motion. The addition of propranolol leads to a small, but significant reduction of the mobility of EGFR along the membrane. As next step we expect that propranolol induces the internalization of the receptor [15]. In order to investigate this on a single receptor level, we screened all CSD plots for longer pauses in mobility. Figure 6 shows an example of a receptor that suddenly got stalled in presence of propranolol. In 6 out of 38 measurements that were performed in presence of propranolol the QD stopped and stayed stationary for at least 10 s (in 5 different experiments and cells). In 4 cases, EGFR continued its motion after the stationary phase. Without propranolol, such stalling was never observed (n = 54). We speculate that this stalling represents the first step in the propranolol induced internalization pathway. Complete internalization, which should show as a disappearance of the QD, was never observed and might have been prevented by the relative large dimensions of the QD (≈ 10 nm diameter).

## Discussion

EGFR has been intensively studied, principally due to its participation in controlling fundamental cellular functions and its role in cancer development. In the absence of EGF propranolol indirectly induces the internalization of EGFR in a dose-time dependent manner [15]. This makes propranolol a highly interesting alternative to control EGFR functioning by lowering the number of the available receptors on the cell surface. 

To gain a better understanding of the events that eventually lead to the internalization process, we studied the effects of propranolol on the mobility of EGFR before the actual internalization took place. By monitoring the mobility of the EGFR along the membrane the effects of propranolol on the interaction of the receptor with the cell cortex and other binding partners and its tendency to aggregate in larger assemblies can be studied. Using single particle tracking of EGFR in HeLa cells we surprisingly found, both with and without the drug, that the receptor shows a pronounced stop-and-go motion; the diffusive path was continuously interrupted by short pauses that lasted for just a few frames. A similar phenomenon was described for EGFR in human epithelial cells [31], who attributed the pauses to the receptor getting stuck in 'nano-domains'. Because our analysis shows that the step-size of the semi-stationary phase is close to that of fixed QDs, this argues for a domain with a very low diffusion coefficient itself (*D* ≈ 0.01 µm^2^/s, table 1). It has been shown that receptors can be transiently trapped by the actin cytoskeleton or by lipid rafts [32], and EGFR was recently reported to form transient clusters on the cell membrane [27]. Trapping of EGFR to clusters is consistent with our observations; the first peak corresponds to the occasional sticking of the observed protein to the cluster and the second peak belongs to the actual diffusion through the membrane between such clusters. In our measurements this clustering accounts for ≈ 20 % of all events and therefore leads to an underestimation of the unrestricted diffusion coefficient. 

In HeLa cells, we calculate an average diffusion coefficient for EGFR monomers of Dcontrol = 0.077 µm^2^/s, which is comparable with values that were reported for EGFR [24,33,34]. Lipids in a synthetic bilayer move with 3.0 µm^2^/s considerably faster [35]. This difference is partly explained by the pauses that are present in the diffusive tracks of EGFR, and also by the larger drag coefficient of the receptor as compared to a lipid. Under the assumption that the diffusivity in membranes follows the Stokes-Einstein relationship the drag of the lipid or receptor should scale with the inverse of its mobility [36].

In a recent study temporal changes in the diffusion coefficient of single EGFR were used to follow the dynamics of dimerization of EGFR [24]. Since dimerization increases the drag on the receptor, it may be possible to distinguish between monomer and dimer states by a difference in their diffusion constant. Indeed, Chung et al. showed within single diffusive tracks a two-fold change of diffusion constant, which they attributed to monomer-dimer transitions of EGFR. Another recent study, where two different coloured labels were used to directly show receptor dimerization, showed however that the dimerization cannot be simply deduced from a temporal change in the diffusion coefficient [25]. In our experiments we also tried to use the change of the diffusion coefficient as an indicator to screen the effects of propranolol on the dimerization rate of EGFR. Despite using similar techniques as in [24] we could not unambiguously observe dimerization events in single diffusive tracks. Although some of our CSD curves (like Figure 2e) show apparent changes in slope during diffusion, the majority of the curves looked like Figure 2f, without obvious transitions between two slopes. The stop-and-go motion of the receptor may be one of the reasons why it is very difficult to use the diffusion coefficient as a sole marker to distinguish monomers from dimers, but also the histogram (Figure 4) shows the presence of only a single mobile population.

The main goal of this study was to test if propranolol affected the mobility of single EGFR before internalization in the absence of ligand. In all cases the mobility of EGFR was dominated by a stop-and-go motion. In presence of the drug we found two effects. The first effect was a 22 % reduction of the average diffusion constant. This reduction is mainly caused by the reduction of the unrestricted diffusion coefficient and not by an increase of the number, or duration, of the pauses. The short pauses show transient sticking or clustering of EGFR and are not affected by propranolol. The reduction of the unrestricted diffusion coefficient can be explained by an increased drag of EGFR through the membrane. From AFM membrane tether extraction experiments we could exclude an increase of the membrane bending rigidity or the membrane interaction with the cell cortex. This suggests that the reduced diffusion coefficient is caused by the binding of EGFR to other binding partners or by clustering of the receptor. The latter option is supported by the finding of Ariotti et al. who reported that the addition of exogenous PA leads to clustering of EGFR [37]. To identify the exact nature of the interactions of EGFR, labelling experiments in which the various possible partners are tagged with different colours will be helpful to identify the players that are involved in regulating the mobility of EGFR. As a second effect of propranolol we found that the receptor gets frequently stalled for longer periods of multiple seconds. This inhibited mobility of the receptor upon drug treatment may signal the entering of the receptor on an endocytic platform, a first step of the internalization process that will lead to the drug induced endocytosis. 

## Materials and Methods

### TIRF microscopy

TIRF imaging was performed on a custom built inverted microscope. All opto-mechanical parts were purchased from Thorlabs GmbH (Germany), except for the following items. The xy stage (Märzhäuser Wetzlar GmbH, Germany) and the PLAPON 60x 1.45NA objective (Olympus, Japan). The sample was illuminated by coupling in a single mode fibre with an output power of 2 mW at 488 nm (Nichia, Japan). The light from the fibre was collimated and then focussed on the back focal plane of the objective using a 100 mm focal length lens. The laser was coupled in the optical path via a dichroic mirror (Di01-R488, Semrock, Rochester, NY, USA) that was placed under the objective. To be able to change the angle of the laser beam coming from the objective, a mirror was placed 100 mm before the laser-focussing lens. Thus a tilting of the mirror results in a lateral translation of the beam in the back focal plane of the objective. The light from the objective was passed through two emission filters in series (595/50 and 630/75; centre wavelength/width) and focussed with a 75 mm focal length tube lens onto an EM-CCD camera (Luca-S, Andor, Ireland), which resulted in a total magnification of 205 nm per pixel. All movies were recorded at a frame rate of 20 Hz. The experiments were carried out at room temperature (25°C).

### Open-top sample holder

To facilitate the addition of propranolol containing medium before the experiments we used a simple open-top sample chamber. The microscope coverslip containing the cells was glued using grease (Apiezon-H, M&I Materials Ltd., UK) onto a microscope slide that had a 10 mm aperture cut out (inset Figure 1A). During the assembly, the cells were kept under medium at all times. The opening in the microscope slide was made using a small sand-blasting device (Renfert GmbH, Germany), after each experiment the slides were cleaned to be reused.

### Preparation of Fab-QDs and tagging single EGFR with the conjugate

To avoid receptor dimerization by the anti-EGFR antibody, only the isolated Fab fragments were used. EGFR–Fab-biotin was produced by pepsin digestion of an anti-EGFR IgG (AB-11; clone 199.12, LabVision) followed by reduction of the resulting F(ab')2 to yield a Fab fragment which binds specifically to the EGFR ectodomain 12–14. The Fab was then conjugated with biotin through the unique -SH at its carboxy-terminal. To prepare anti-EGFR–Fab-QD conjugates, 1 ml of a solution of 0.7 nM anti-EGFR–Fab-biotin in DMEM was added drop-wise to 1 ml of 2 nM QDs coated with streptavidin (CdSe, QD605, Invitrogen). Thus, on average every QD carried less than 1 anti-EGFR–Fab-biotin. QD605 are exited at 488 nm wavelength and emit photons of lower energy at ≈ 605 nm.

### Cells

HeLa cells (250.000 cells) were cultured on glass coverslips. Cell monolayers (70% confluent) were serum starved for 2 hours before being incubated with 500 µl of the final anti EGFR–Fab-QD solution at 25 °C for 2 min. Cells were then washed twice with PBS at 25 °C, placed in serum-free medium (DMEM + 25 mM Hepes pH 7.4), and mounted into the sample holder. For the experiments in presence of the drug, 150 mM of propranolol was present in the serum-free medium.

### Tracking algorithm

To determine the position of single QDs with sub-pixel accuracy, the centre of the imaged spot had to determined. To estimate the centre of the point spread function we used a 2D-Centroid algorithm similar to the one described by 26. Basically, the centre of intensity in a pre-selected region in the image is calculated. The routine was written as a plug-in for ImageJ and performed the following steps:

1 Select a QD in the first frame and select a 2 x 2 µm region around it, which does not contain any other QDs
2 Proceed to next frame, calculate the new centre of intensity and re-centre the selected region.
3 Repeat step 2 until last frame.

### Fitting of the diffusion step-size histogram

To obtain an estimate for the unrestricted diffusion coefficient of the receptor we fitted a probability density function (eq. 1) to the step-size histogram (see table 1 for the fit values). The first peak we attribute to the semi-stationary 'slow' QDs, and the second peak to the unrestricted 'fast' moving QDs. Both peaks are fitted with a step-size distribution of a 2D diffusion process [38]. *Dslow and Dfast* are the diffusion coefficients of the respective peaks, *x* the step-size and Δ*t* is the time between frames (0.05 s). *C* gives the proportional contribution of the semi-stationary QDs to the histogram. Before fitting, the total surface area of the histograms was set to 1 by rescaling the height of the bars.

$$PDF(x)=C*\underset{\text{'slow' peak}}{\underbrace{\frac{x}{2D_{slow}\Delta_{t}}\exp\left(-\frac{x^{2}}{4D_{slow}\Delta_{t}}\right)}}+(1-C)*\underset{\text{'fast' peak}}{\underbrace{\frac{x}{2D_{fast}\Delta_{t}}\exp\left(-\frac{x^{2}}{4D_{fast}\Delta_{t}}\right)}}$$ (1)

### Atomic force microscopy

The membrane tether extraction measurements were performed on an Asylum MFP3D AFM (Asylum Research, CA, USA) that was placed onto a bright-field illuminated inverted optical microscope equipped with a 60x 1.49 N.A. objective. First, a cell was selected with the optical microscope and the AFM tip was positioned above the cell body but away from the nucleus. Then the tip was brought down to touch the cell at a force of 0.3 nN. After 2 seconds the tip was pulled away for a distance of 8 µm at a relatively low pulling speed of 1 µm/s. In about 50% of all curves a membrane tether was pulled off the cell by the AFM tip, which was visible as constant force plateau when the tip was retracted. Most tethers could not be extracted for the full 8 µm but ruptured, which is visible in the force curves as a sudden jump to zero force (inset Figure 5). To obtain the tether force we analysed only those curves in which the force before and after the rupture event was constant for at least 500 nm of pulling. The tether force was quantified by the difference of the average force of the 500 nm before and of the 500 nm after the rupture. For the experiments we used soft cantilevers (BL150, 30 x 60 µm, Olympus, Japan) with a calibrated spring constant of ≈ 0.03 N/m. To minimize the effects of calibration errors we used 4 different cantilevers, each of which for both the control and the experiments with propranolol. The experiments were performed at 24°C.
